# Effects of Calomel on the Teeth

**Published:** 1848-07

**Authors:** J. Taylor

**Affiliations:** Cin. Ed.


### EFFECTS OF CALOMEL ON THE TEETH.

BY J. TAYLOR, M. D. D. D. S.—CIN. ED.

There is no opinion more common, or more firmly believed by the mass of the community, in relation to the decay of the Teeth, than that Calomel (I shall use the term most universally understood by those not acquainted with medical technicalities) acts directly and injuriously upon the dental organs. The belief is so prevalent, and caught at so readily by many, that we can scarcely ascertain whether they regard it as necessary that this substance actually come in contact with the teeth or not. Some appear to regard this as necessary, but that the slightest touch is sufficient; while others regard it as so searching in its operation, and so poisonous in its effects, that if it once passes into the stomach, even in the smallest quantity, that muscle, blood, nerve, ligament, tender, cartilage and bone, all receive their due portion, and feel its poisoning and blightning influence. The joints stiffen, and the hardest and most dense organ of the system crumble and decay beneath its powerful action.

How often we hear the doleful cry, Calomel ruined my teeth; when, upon inquiry, we find they have never taken more than one small portion, and that years gone by, and its effects on the gums had never been apparent.

These views are often entertained by those whose knowledge of chemistry, upon slight reflection, would make them sensible of their error. It appears to be far easier to fall in with a common and popular prejudice, than to investigate its claims to our belief. This is often the case when but little effort would unravel the whole.

The basis of the enamel as well as bone of the teeth, is phosphate of lime. Calomel is a compound substance—designated, until recently, by the chemical term of Sub-muriate Hydragyri—the term implies a deficiency of acid; hence, in Henry's Chemistry, we find the composition set down as follows:—100 mercury; 4.16 oxygen, 27.39 muriatic acid. We will now go a little further, and trace out the respective affinities of the phosphoric and muriatic acids for the lime and murcury.

Henry has placed the affinity of the different acids for murcury, in the following order: Gallic, Muratic, Oxalic, Succinic, Arsenic, and then Phosphoric Acid.

The same author has placed the affinity of the different acids for lime in the following order: Oxalic, Sulphuric, Tartaric, Succinic, Phosphoric, Mucic, Nitric, and then Muriatic Acid.

By a careful study of the above table—keeping in view the laws of chemical affinity, we must come to the conclusion that Calomel, in its pure state, exerts no injurious effects on the teeth; to effect this, say in the mouth by its local application, it must first undergo a chemical change—its acid be disengaged from the metal, and permitted thus in a free state, or in a diluted form, (for the saliva would dilute it,) to act upon the teeth. It must be remembered we are now considering its local effect. We can hardly conceive it possible, even if a chemical change could take place in the mouth, that a sufficient amount, by proper administration, should even remain about the teeth, to thus prove a source of injury. To illustrate more forcibly, we will take an article almost unversally used at every meal, and by every individual, and often too as a dentrifice. We allude to common table salt. This is the muriate of soda, and, according to Dr. Marcet, is composed as follows: Muriatic acid, 46; Soda, 54. Here we have an article in general use containing a far larger amount of acid than calomel; yet no one attributes any injurious effects to its use, and some, indeed, recommend it as an excellent dentrifice for the teeth and gums.

We think, however, that most of the salts and Alkalies are very properly left out of dentrifices by scientific dentists.

It would be useless to pursue this part of the subject any further. For taking the known laws of chemical action for our guide, we will find that all the sulphates and the tartrates, which are as often used as remedial agents as the calomel, would prove far more injurious to the dental organs. We take it for granted, that if any of the salts act injuriously, it would be those holding in combination acids having a greater affinity to the lime than phosphoric.

From what has been said, we think it apparent that there is no just ground for the very prevalent belief, that calomel, by actual contact with the dental organs, can produce that injury which the mass of the community suppose.

Let us, however, take a hasty glance at its secondary effect. In its action as a direct purgative, none would suppose that the dental organs would be in the slightest manner acted upon. But in its action on the glands of the mouth, as in ptyalism, a very different state of things takes place. That secretion called the saliva, which is constantly thrown into the mouth, and which continually surrounds and moistens the teeth, is now more or less changed; and if that change should be such in its chemical nature as to produce a solvent sufficiently powerful to act upon the enamel and bony structure, then destruction of the organs to some extent would necessarily be the result.

Saliva, in a normal state, cannot, as a matter of course, be destructive to the dental organs. The God of nature has not so arranged the beautiful machinery of our complex system, that the healthy action of any one organ may be injurious to the proper action of another. In health, all work together, each performing their proper function, and each more or less dependent upon some other; but derange one, and it brings in its train the deranged and morbid action of another.

The healthful secretion of saliva is beneficial to the preservation of the teeth. Perhaps this proposition may not be generally accepted; yet I would reaffirm, that "the healthful secretion of saliva is essential to the preservation of the teeth." When not vitiated, it acts as the most rapid and certain neutralizer of acids, or any carroding substance that may be taken into the mouth.—Nature furnishes, in her operation on the system, none other so well adapted for this purpose. The amount supplied appears to be proportionate to the necessity of the case: and we believe that, were it not for this secretion, so well supplied, and silently and constantly acting upon chemical agents, which almost daily come in contact with the dental organs, that our teeth would soon be destroyed. The inquiry, however, next is, to what extent is the saliva changed, when the system is under the full impression of calomel? We will first give the constituent of the healthy secretion. Dr. Wright gives the following:

| | |
|---|---|
| Water, - - - - - - - - | 988.1 |
| Ptyalin, - - - - - | 1.8 |
| Fatty acid, - - - - - - | 5 |
| Chlorides of Sodium and Potassium, - | 1.4 |
| Albumen with Soda, - - - - | 9 |
| Phosphate of lime, - - - - | 6 |
| Albuminate of Soda, - - - - - | 8 |
| Lactates of Potash and Soda, - - - | 7 |
| Sulphocyanide of Potassium, - - - | 9 |
| Soda, - - - - - - - - - | 5 |
| Mucus with Ptyalin, - - - | 2.6 |

We find, however, that the saliva varies more or less in its constituents, if the analysis is made immediately after a meal, or during a fast, yet not so as to effect materially the chemical properties of this fluid, particularly as it regards any action on the teeth. We find in the above table a mere fraction of fatty acid, yet of too small a quantity to make an impression on the teeth, even if the acid had a greater affinity to the lime than the phosphoric. We shall give, perhaps, as clear and full a view of the change which this fluid undergoes, by its passing into a morbid state, by giving a lengthy extract from Simon's Chemistry of Man on this subject.

This author remarks, that "the saliva becomes affected in various morbid conditions of the system; but the nature of the changes that it undergoes, has not hitherto been sufficiently studied. Morbid saliva sometimes contains a free acid; this is most commonly the lactic acid; but, in some cases, acetic acid is likewise present. The acid reaction may be at once detected by test paper; while normal saliva communicates a blue tint to red litmus paper, this, on the contrary, reddens blue paper. I have frequently seen the saliva acid in acute rheumatism, and in cases of *salivation.* According to Donne, the saliva has an acid reaction in all cases of irritation and inflammation of the stomach, in pleuritis, encephalitis, intermittent fever, acute rheumatism, uterine affections, and amenorrhœa. Brugnatelli detected oxalic acid in the saliva of a phthisical patient. The secretion of saliva is sometimes increased to an extraordinary degree, constituting salivation; in such cases, the chemical characters of the saliva are also more or less affected. In a specimen of saliva forwarded to me for examination, which was obtained from a patient who had just terminated a course of mercury of some weeks duration, I observed an acid reaction, arising from the presence of free acetic acid. It was very viscid, of a yellow color, and possessed a sickly disagreeable acid smell. It contained no mercury. After evaporation to dryness, all the acid reaction had disappeared;—thus showing that it contained no free lactic acid. This saliva contained a very large quantity of semi-fluid fat,—a considerable amount of albumen, and traces of caseous matter. Under the microscope, an immense number of fat-vessicles were seen, some epithilium-cells, and a very few partially-destroyed saliva corpuscles. 1000 parts of this saliva were composed of

| | Analysis 59 |
|---|---|
| Water, - - - - - - - - - - | 974.12 |
| Solid constituents, - - - - - - - | 25.88 |
| Yellow viscid fat, - - - - - - - | 6–94 |
| Ptyalin, with extractive matter and traces of casein, | 3–60 |
| Alcohol---extract with salts, - - - | 7--57 |
| Albumen, - - - - - - - - - | 7--77 |

The salts consisted of a largely preponderating amount of the chlorides of sodium and potassium, associated with the lactates of soda and potash, and with a small quantity of the earthy phosphates. On contrasting this saliva with the normal fluid, we are struck with its large amount of solid constituents, arising not from any increase of the ptyalin, but of the fat, the extractive matters, the albumen, and the salts."

From the above extract, we find that this secretion during salivation undergoes some considerable changes, and sometimes to a sufficient degree to have its appropriate acid effect on litmus paper. We presume it would be difficult to determine why the same acids are not always found in the saliva, when effected by this same medicine; yet we find at times the presence of the lactic, the acetic, and in a phthisical patient the oxalic. The two latter of these possess a powerful affinity for the lime of the tooth; and we should be disposed to think that whenever their presence was of sufficient amount to redden blue litmus paper, that a perceptable effect in time would be observed upon the teeth. It may be considered probable that, under certain conditions, the saliva may, during salivation, be so changed as to produce an injurious effect on the dental organs. The amount of injury, we presume, might be calculated in proportion to the amount of acid, particularly the acetic acid, which might be found therein, and the duration of the salivation. The same thing, however, may take place from a vitiated condition of the saliva from other causes, some of which have already been enumerated. Taking this view of the subject, and carrying out the train of thought suggested by the changes wrought in the saliva by disease, and the various remedial agents used for the cure of diseases, how important a proper understanding of this whole subject becomes to the dental practitioner.—How important that the condition of the mouth, the quality of the saliva, &c., should be well ascertained, before prescribing a wash or dentrifice. If we mistake not, here is just where much is yet to be done for dental science. We have seen hundreds of mouths, the teeth of which were in much the same condition as could be produced in a short time by dilute acetic or sulphuric acid, When we see the same nature of decay existing thus; as we may produce, what is the indication? we would say, unhesitatingly, not a dentrifice in which cream of tartar enters as a component. But this subject would form an article of itself sufficiently long for the Register, and we may at another time take it up in detail.

We shall, in conclusion, merely allude to the fact, that should salivation take place during the formation of the permanent teeth, when they are passing from their pulpy to their osseus structure, that the bone may not then be secreted and properly formed; yet we have seen teeth apparently in every respect perfect in form and organization—formed, to some extent at least, during severe salivation; and we cannot say they appeared to be any more subject to disease than if such had not been the case.

